# Supplementary material for: An Environmentally Friendly Supramolecular Glue Developed from Natural 3,4-Dihydroxybenzaldehyde
Source: Polymers (Basel). 2022 Feb 25;14(5):916. doi: 10.3390/polym14050916 (PMC8912294; doi:10.3390/polym14050916)
Supplement: Supplementary file 1 [file polymers-14-00916-s001.zip › polymers-1597121-supplementary.pdf]

An environmental-friendly supramolecular glue developed from natural 3, 4-dihydroxybenzaldehyde

Hui Wang, Xin Du, Yuanyuan Liu, Xiangjiang Liu, Ailing Sun, Liuhe Wei*, Yuhan Li*

College of Chemistry and Green Catalysis Center, Zhengzhou University, Zhengzhou Key Laboratory of Elastic Sealing Materials, Zhengzhou 450001, P.R. China

E-mail: weiliuhe@zzu.edu.cn (Liuhe Wei); liyuhan@zzu.edu.cn (Yuhan Li)

Supplementary Figures and Tables

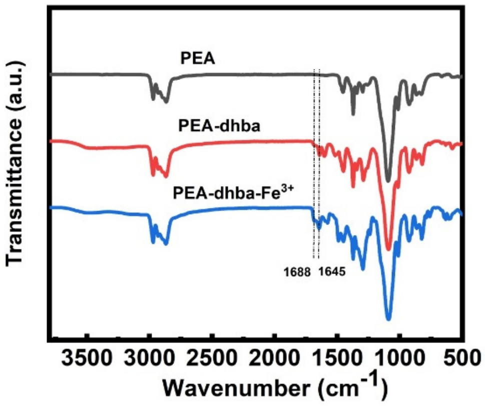

Figure S1. FTIR spectra of PEA, PEA-dhba and PEA-dhba-Fe^{3+}.

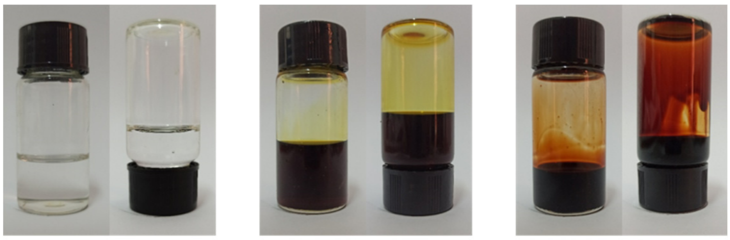

Figure S2. Photographs showing PEA, PEA-dhba and PEA-dhba-Fe^{3+} solutions.

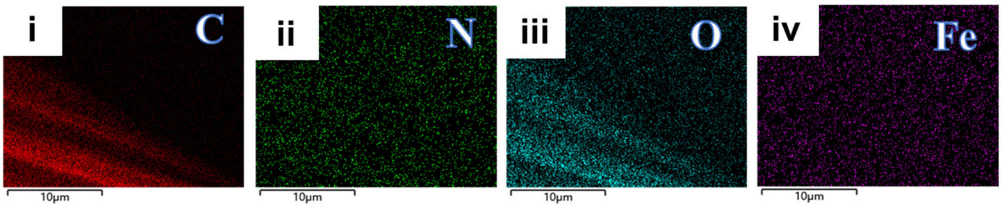

Figure S3. EDS mapping results of dried PEA-dhba-Fe3^{+} bulk.

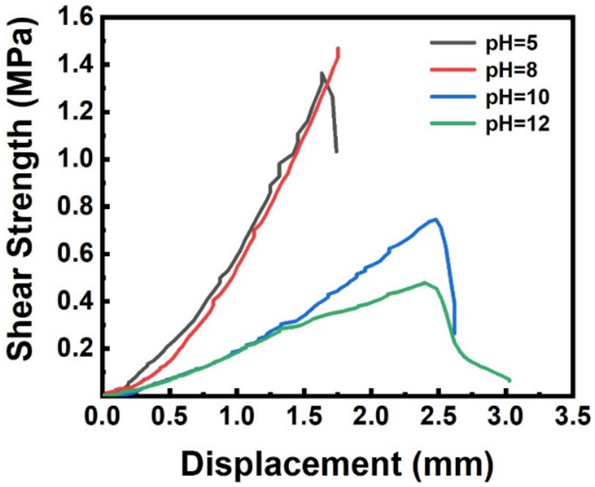

Figure S4. Shear strength plotted to displacement of PEA-dhba-Fe3+ bulk obtained from solutions with various pH values, the substrate utilized was aluminum.

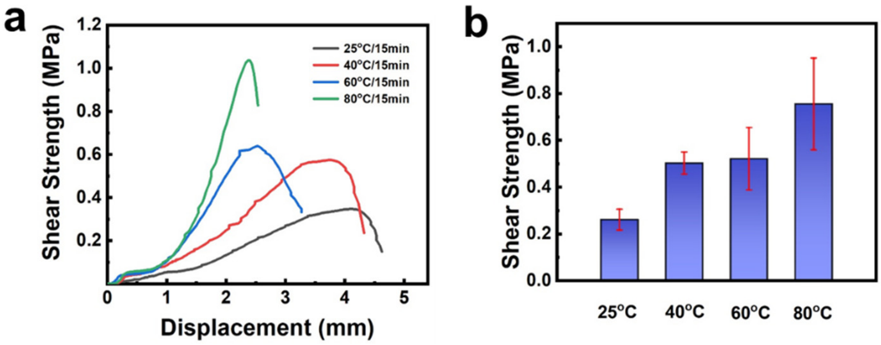

Figure S5. (a) Shear strength plotted to displacement of PEA-dhba upon various drying temperature for 15 minutes and (b) corresponding shear strength comparison.

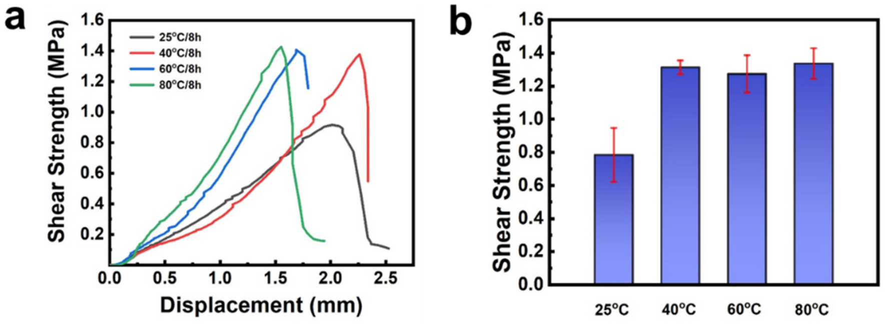

Figure S6. (a) Shear strength plotted to displacement of PEA-dhba upon various drying temperature for 8 hours and (b) corresponding shear strength comparison.

Table S1. Characteristic peak assignments of PEA-dhba-Fe^{3+}.

**Wavenumber (cm^{-1})**	**Assignments**
3500	H-bonded ν (OH)
2942	$\nu_a(CH_2)$
2911	$\nu_a(CH_2)$
2854	ν_a (CH_2)
2792	ν_s (CH_2)
1645	ν_s(-C=N-)
1604+1498	benzene ring
1291	ν(-C=C-)
1089	ν (C-O-C)

Table S2. Theoretical bare phenolic groups corresponding to mono-, bis-, and tris-complexation modes at various molar feed ratio of Fe^{3+} : dhba.

Mode	1:15	1:10	1:5	1:3
Mono-	0.037	0.036	0.032	0.027
Bis-	0.035	0.032	0.024	0.013
Tris-	0.032	0.028	0.016	0

Note: calculated based on the original total amount of phenolic groups equaling to 0.04 mol.
